# Transdiagnostic Tailored Internet- and Mobile-Based Guided Treatment for Major Depressive Disorder and Comorbid Anxiety: Study Protocol of a Randomized Controlled Trial

**DOI:** 10.3389/fpsyt.2018.00274

**Published:** 2018-07-04

**Authors:** Kiona K. Weisel, Anna-Carlotta Zarski, Thomas Berger, Michael P. Schaub, Tobias Krieger, Christian T. Moser, Matthias Berking, David D. Ebert

**Affiliations:** ^1^Department of Clinical Psychology and Psychotherapy, Friedrich-Alexander-Universität Erlangen-Nürnberg, Erlangen, Germany; ^2^Institute of Psychology, Leuphana University of Lüneburg, Lüneburg, Germany; ^3^Department of Clinical Psychology and Psychotherapy, University of Bern, Bern, Switzerland; ^4^Swiss Research Institute for Public Health and Addiction ISGF, University of Zurich, Zurich, Switzerland

**Keywords:** depression, anxiety, comorbidity, transdiagnostic, tailored, internet intervention, randomized controlled trial, guided self-help

## Abstract

**Introduction:** Depression is highly prevalent and often accompanied by comorbid anxiety disorder. Internet-based interventions have shown to be one effective treatment modality; however, comorbidities are often not targeted. Transdiagnostic tailored internet-and mobile-based interventions (IMIs) might be promising to overcome such issues.

**Aim:** This study aims to evaluate the efficacy, moderators, and cost-effectiveness of a transdiagnostic tailored internet- and mobile-based guided intervention for depression and comorbid anxiety in individuals with major depressive disorder (MDD).

**Method:** Two-hundred participants with MDD will be randomly assigned to an 8-week guided self-help internet intervention (IC) or a 6-month wait-list control group (WLC). Participants of the IC will receive weekly content-focused feedback on module completion as well as monitored adherence reminders from an eCoach. The primary outcome is clinician-rated depression severity (QIDS-C) at post-assessment assessed by diagnostic raters blind to study condition. Secondary outcomes include, e.g., change in diagnostic status (MDD and anxiety disorders), remission and response rates, disorder symptom severity, health related quality of life, incongruence related to needs and values, and behavioral activation. Assessments will take place at baseline (T1), post-assessment (T2), 6-month follow-up (T3), and 12-month follow-up in the IC. Data will be analyzed on an intention-to-treat basis and per protocol. A large number of a priori defined moderators of treatment outcome will be assessed at baseline and tested in predicting treatment outcome. Cost-effectiveness will be evaluated from a societal perspective.

**Discussion:** The present study will provide evidence on the efficacy, potential cost-effectiveness, and moderators of a transdiagnostic tailored guided internet- and mobile-based treatment protocol.

Trial Registration: German Register of Clinical Studies DRKS00011690 (https://www.drks.de/drks_web/).

## Introduction

Depression is one of the most prevalent common mental disorders (CMDs) worldwide (1, 2), with a range of adverse personal and societal consequences (3–5). Prevalence rates of major depressive disorder (MDD) in the US and Germany are estimated at ~19% for lifetime prevalence and range from 6% to 12% for 12-month prevalence (1, 2, 6). Reducing the prevalence rates and disease burden is an important issue from a public health perspective (6). Depressive disorder is associated with negative physical and mental health outcomes such as reduced quality of life, suicide ideation, chronic medical conditions, disability, noncompliance with medical treatment, and heightened mortality risk (7–13).

Individuals suffering from MDD have an increased likelihood of comorbidity such as suffering from another CMD (14–16). Comorbidity is estimated to range from 40% to 80% in both clinical and epidemiological studies (14, 17). Individuals suffering from MDD often also suffer from anxiety disorder (AD) as found in an epidemiological study with an estimate of 53% (18). Comorbidity negatively influences daily functioning, is linked to greater severity and chronicity of disorder course, and associated with higher mortality in medical conditions such as heart disease (8, 19).

Even though effective treatment modalities exist for MDD and for ADs (20–22), there is still a large unmet need for treatment. Treatment barriers can be categorized as structural, e.g., restricted accessibility to treatment and availability thereof, and communities with high mental health stigma might prevent individuals from seeking help, or individual and attitudinal treatment barriers such as physical impairment, time constraints, unaffordable treatment costs, preference for other types of treatment, or preference for self-help (23, 24).

One modality of treatment delivery is through internet- and mobile-based interventions (IMIs) which can help overcome some traditional treatment barriers and generally facilitate treatment utilization. Advantages include (1) anonymity, (2) treatment flexibility, (3) low threshold access, (4) fostering self-efficacy, (5) cost-population scalability, and (6) standardization. A fair amount of studies have shown internet interventions to be effective in treating symptoms of depression and anxiety (25–28). A review examining treatment for depression considered five studies with 429 participants comparing face-to-face and guided internet-based CBT (iCBT) (25). Meta-analytic summary statistics found average effect sizes Hedges' *g* = 0.12 (95% CI: −0.06–0.30) in favor of guided iCBT which indicates both type of treatments to be comparable. Another extensive meta-analysis explored the effects of therapist-supported iCBT compared to wait-list, unguided CBT, or face-to-face CBT on remission of anxiety disorder diagnosis and reduction of anxiety symptoms in adults (28). After screening by two independent researchers, 30 studies were included which showed iCBT to be efficacious in reducing anxiety symptoms. Overall, meta-analytic evidence found guided iCBT to produce comparable overall effects to face-to-face CBT in treating depression and anxiety disorders (28).

However, utilizing internet interventions for the treatment of mental disorders can also pose some challenges, such as low treatment commitment, incompatibleness of personal characteristics and treatment modality self-help, and missing or limited individualization options which may lead to higher dropout rates and decreased overall efficacy (29–31). To overcome some of these issues, their impact must be carefully considered during the study design phase and strategies to diminish negative effects should be incorporated, e.g., adherence monitoring, therapeutic contact through guidance, and individual tailoring.

Recent systematic reviews indicate that internet-based interventions can also be a cost-effective mean to treat depression and anxiety, when compared to wait-list, treatment-as-usual (TAU), or unguided iCBT (32, 33).

However, despite the high comorbidity rates of MDD and ADs, it is currently recommended and standard procedure to treat mental health disorders separately with disorder-specific interventions (DSIs), e.g., through indicative CBT (34, 35). Even though many DSIs have been developed, evaluated and shown to be effective in randomized controlled trials (RCTs) (20–22), there seem to be some limitations to treating disorders in isolation without targeting comorbidity. Potential risks include that DSIs might dismiss comorbid symptoms and comorbid conditions could worsen, comorbidities could negatively influence treatment response in DSIs, participants might need prolonged treatment or several separate treatment procedures, and due to treatment costs affected individuals might not be able to afford all the necessary treatments (15, 19, 36, 37).

Therefore, transdiagnostic treatment concepts, treating at least two disorders simultaneously, might help overcome some issues of DSIs (37). Transdiagnostic interventions can be designed in different manners by identifying and addressing common risk factors including etiological and maintaining factors, common underlying behavioral mechanisms of MDD and AD, and overlapping symptom profiles (38–40).

There is meta-analytic evidence that transdiagnostic computerized CBT programs can result in large effect sizes for depression (*g* = 0.84) and medium effect sizes for anxiety (*g* = 0.78) (41). Moreover, a recent meta-analysis of transdiagnostic psychological treatments for anxiety and depression found manualized transdiagnostic interventions, delivered face-to-face or over the internet to be effective with large effects for depression (*g* = 0.91) and anxiety (*g* = 0.85) (42). However, heterogeneity was high indicating significant differences in treatment effects across studies. More high quality RCTs are needed to investigate the efficacy of transdiagnostic internet-based treatment protocols.

Comparing transdiagnostic concepts to DSIs, recent meta-analytic exploration indicates transdiagnostic internet-based treatments could be as effective for reducing anxiety symptoms and potentially superior in reducing symptoms of comorbid depression compared to DSIs which focused solely on anxiety (42, 43).

It is possible that the model “one size fits all” commonly applied in DSIs and in manualized transdiagnostic interventions might not be effective for all participants. A method to overcome limitations related to standardization is by applying individual tailoring techniques of treatment content. In these instances, the treatment protocol is not standardized for all participants, but can rather be adapted individually based on different criteria, such as participants symptom profiles, stage of motivation, preference, needs, characteristics, or clinician recommendation (37, 44, 45). Even in standardized face-to-face therapy, individual tailoring can be an integral part of the treatment expressed by, e.g., vocal inflection, personal circumstances, type of psychoeducational model, and therapists' work experience. These elements often cannot be translated directly to internet interventions which is why it is important to include other elements of individual tailoring to personalize internet-based psychological treatment.

A meta-analysis investigated the efficacy of internet-delivered transdiagnostic and tailored CBT for comorbid anxiety and depression and found controlled moderate to large effects for both depression and anxiety (anxiety: *g* = 0.82, 95% CI: 0.58–1.05, depression: *g* = 0.79, 95% CI: 0.59–1.00) compared to control groups which included wait-list and active wait-list on the basis of 19 studies including 2952 participants (37). However, there are generally few RCTs investigating the effects of transdiagnostic treatment and individual tailoring.

Another issue which has not been explored sufficiently to date are differential treatment effects of IMIs, e.g., who profits from participation in such interventions and who does not. Trials designed to investigate overall treatment effects are generally underpowered to adequately investigate efficacy in subgroups, as approximately four times as many participants are needed to detect an interaction effect between a moderator and the treatment condition compared to the primary effect (46). Furthermore, moderation analyses are often not planned a priori so that it is unlikely relevant moderators are additionally added to the study assessments. However, it is important to include a broad range of potential moderators, to power, and to test for moderation, as identifying moderators of treatment outcome can help better understand heterogeneity of treatment response (47).

The aim of the present study is to evaluate the efficacy, moderators, and cost-effectiveness of a transdiagnostic individual participant tailored internet-based mobile-supported guided intervention for depression and anxiety in individuals with MDD with and without comorbid anxiety.

## Methods

### Design

A two-armed RCT will be conducted to compare the intervention condition (IC) ICare Stimmung with a wait-list control group (WLC). Individuals assigned to the WLC will receive access to the intervention 6 months after study begin. They can contact the study administration at any time through the messaging function on the platform or by email. They can seek other treatment after study uptake and they additionally receive an information sheet detailing psychological treatment options upon high symptom severity assessed in the diagnostic interview or upon contacting the study administration team.

**Figure 1:**
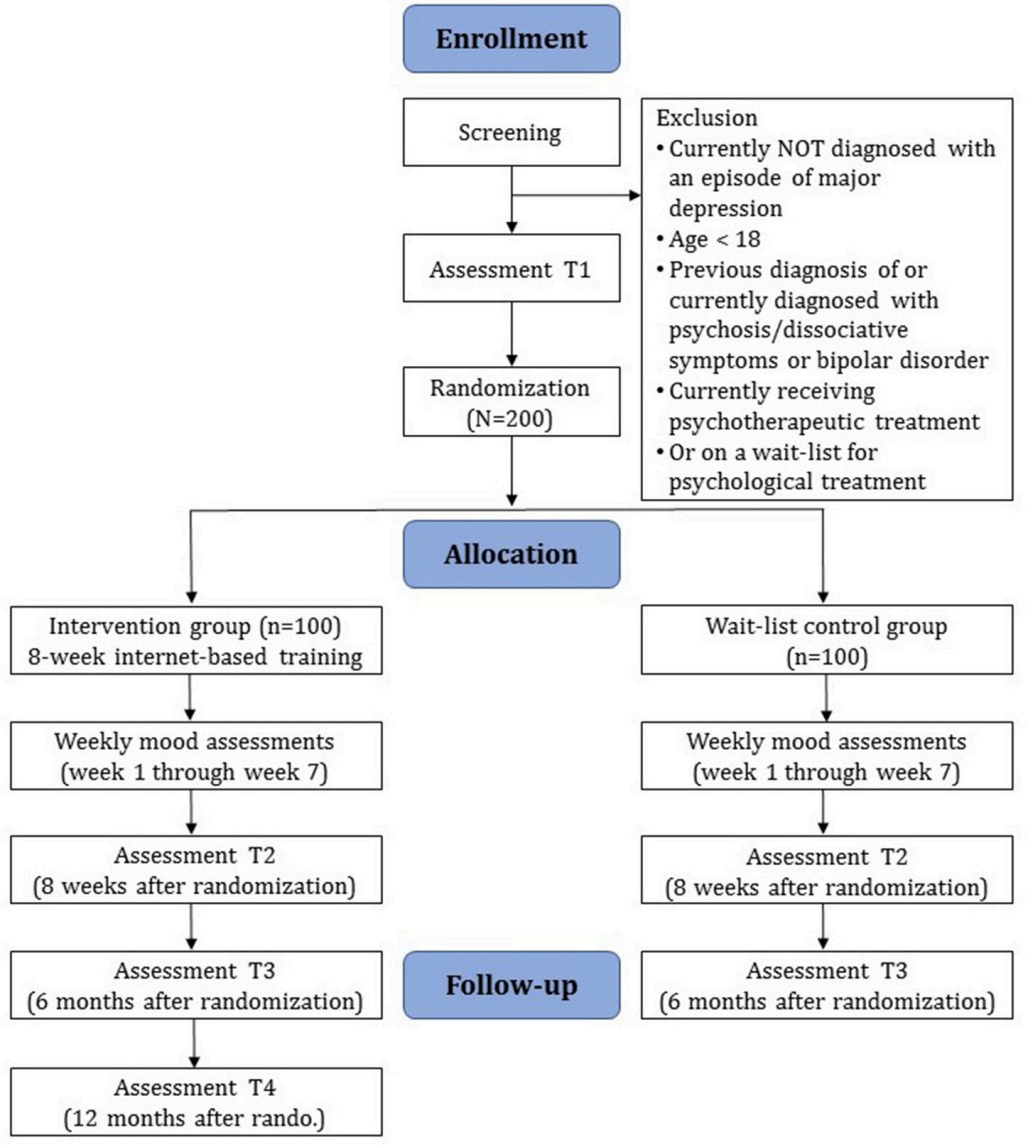
Study flow.

Diagnostic interviews and self-report assessments will take place at baseline (T1), post-assessment (T2), and at 6-month follow-up (T3) (see Figure 1 for a detailed overview). There will also be weekly mood assessments during the first 7 weeks of the study phase. Diagnostic interviews will be conducted via telephone. Self-report data will be collected using a secure online-based assessment system (AES, 256-bit encrypted). All procedures involved in the study will be consistent with the generally accepted standards of ethical practice. The study was approved by the Friedrich-Alexander-Universität Erlangen-Nürnberg ethics committee (No. 144_16B). The trial is registered in the German Clinical Trial Registry under DRKS00011690 (https://www.drks.de/drks_web/).

### Participants and procedure

#### Inclusion and exclusion criteria

We will include (1) adults (≥18 years), (2) with a diagnosis of MDD according to DSM-IV criteria assessed by diagnostic raters using the Mini international neuropsychiatric interview (MINI) (48), (3) internet access, (4) a valid email address, (5) sufficient skills in the German language in reading and writing, and (6) who agreed to the terms of this trial by providing signed informed consent. Individuals are informed that they can discontinue the study at any point without any consequences. Individuals will be excluded who (1) have a history of manic/hypomanic episodes, (2) or psychotic disorder, (3) are currently receiving psychotherapy for MDD or anxiety, (4) are on a wait-list for psychotherapeutic treatment, or (5) received treatment in the previous 6 months, and (6) show a notable suicidal risk in the diagnostic interview. The trial is open to all German speaking participants residing in Germany, Switzerland, or Austria.

#### Recruitment

Recruitment will take place primarily over German and Swiss health insurance companies who advertise the study on their website and in members' magazines. Individuals can take part in the study no matter their insurance status or company. We will also advertise via Facebook and targeted advertising, the study website (www.icareprevent.com), and open recruitment strategies, e.g., radio segments, print media, and social media. The study website provides information on the online treatment and study procedure. This recruitment aims to mimic a public health approach of mental health treatment, in which access to treatment is not limited by face-to-face gatekeepers. Recruitment begin was May 2017. Interested individuals can sign up by providing their email address and a name or pseudonym on the study website or by contacting the study administration directly via email. They are advised that they can create a new email for study participation. Primary recruitment is for *ICare Prevent*, a study on transdiagnostic prevention of depression and anxiety for a subclinical population. Individuals who receive a diagnosis of MDD in the screening process will be excluded from the *ICare Prevent* trial and included in the present study.

#### Assessment of eligibility and randomization

Individuals interested in study participation will receive a detailed email with information on the study procedure. The applicants are then asked to complete an online screening containing question on demographic information, current state and history of mental health, and experience with psychotherapeutic treatment. Individuals who show symptoms of depression (CES-D ≥ 16) and do not fulfill any of the exclusion criteria will receive detailed study information and are requested to acknowledge and sign the consent form. The signed consent form can be sent by postal mail or email. Following informed consent, diagnostic interviews will be conducted by trained diagnostic assessors. Individuals who fulfill all inclusion criteria and no exclusion criteria proceed to complete baseline assessments. Once these are completed, individuals are included in the study and randomized to one of the study conditions, the guided intervention (IC) or the wait-list control (WLC) group.

Randomization will take place at an individual level by an independent researcher not otherwise involved in the study administration. A randomization list is created in a 1:1 ratio based on sequences of 8 per block using an automated computer-based random integer generator (randlist). Until randomization is completed, allocation will be concealed from participants, researchers involved in study administration, and eCoaches. eCoaches are psychologists or psychotherapists in training, licensed psychotherapists, or trained masters' level psychology students providing support to the participants during the duration of the online training. Outcome assessors will remain blind throughout the study. After randomization, participants will be informed of the outcome, and participants in the IC will receive immediate access to the online treatment while participants randomized to the WLC will receive login access to the training 6 months after study intake.

#### Participant safety

Individuals who show symptoms of heightened suicidality will be treated according to a standardized procedure, e.g., during the diagnostic telephone interview individuals will be excluded from study participation and strongly advised to seek help and support from their general practitioner or local psychiatric emergency room. All relevant information containing emergency contact numbers will be additionally sent via email. This procedure will also be followed should participants show symptoms of suicidality throughout the study phase. Additionally, participants already included in the study are contacted by an experienced clinician who will determine suicidality symptom severity and discuss next steps concerning their mental health.

### Intervention development and content

The intervention was developed in an expert-driven manner (49). After the initial development phase, the intervention was tested in a small-scale alpha-testing with a group of students. Additionally, four qualitative interviews with students who had completed the intervention were conducted. The collected data was used to make minor improvements and modifications to the intervention content.

The main sessions cover the following topics: behavioral activation: reducing incongruence, behavioral activation: overcoming difficulties and pleasant activity scheduling, psychoeducation on depression and anxiety, cognitive restructuring, problem solving or exposure, and planning for the future. Individual participant tailoring is promoted by providing participants with frequent choices on intervention topics and treatment preferences. In addition to transdiagnostic elements, they can further choose to focus on depression, anxiety, or both disorders, and work through various elective modules on different topics: rumination and worries, acceptance, relaxation, reducing alcohol, self-worth, perfectionism, appreciation and gratefulness, and sleep. See Table 1 for an overview of the sessions and elective modules.

**Table 1:** *ICare Stimmung* training.

**Sessions**	**Elective modules (sessions 2–7)**
1 Behavioral activation: reducing incongruence	
2 Behavioral activation: overcoming difficulties and pleasant activity scheduling	Rumination & worries, acceptance, relaxation, reducing alcohol, self-worth, perfectionism, appreciation and gratefulness, sleep
3 Psychoeducation	
4 Cognitive restructuring	
5 Problem solving I or exposure I	
6 Problem solving II or exposure II	
7 Plan for the future	
8 Booster session	

Elective modules can freely be chosen based on participant preference throughout the intervention. Furthermore, participants can opt to receive tiny tasks which consist of short motivational notifications and small transfer tasks sent throughout each day during the intervention phase through the app. Furthermore, the intervention strongly relies on content tailoring which engages the participant to make real-time choices among various response options by providing immediate content based on participant preference or need. Participants can choose between two versions of the tiny tasks, the light version (three notifications per day), intensive version (five notifications per day), or they can choose not to receive notifications. The tiny tasks are sent through an additional app component which participants can install on their smartphone. They can also use the app for their diary entries.

The intervention content consists of texts, testimonials, audios, short educational video clips, and interactive elements such as quizzes. To foster self-efficacy and stabilize the long-term learning process, participants are instructed from the beginning to integrate and practice newly acquired strategies in their daily life, e.g., through scheduling individualized weekly activity plans, using online diaries, and receiving daily tiny tasks. At the beginning of each session, participants review the previous session content and evaluate how the practicing of homework worked. Sessions are estimated at ~60 min and participants are advised to do one session per week and a maximum of two. Therefore, the training is supposed to last 4–7 weeks. Responsive web-design enables the training to be completed on any kind of internet-ready device. The read-aloud function allows participants to follow the intervention also through narration.

#### Behavioral activation: reducing incongruence (session 1)

Participants are introduced to core functionalities of the training in the first session, such as testimonials, diaries, videos, and the tiny tasks sent through the app. The first session aims at teaching techniques to reduce incongruence between individual perceptions and goals and thereby enhance psychological well-being. This concept is based on the consistency theory by Grawe (50). Participants are encouraged to explore their personal needs and values and work through ways to align their behavior and strengthen the corresponding life areas. Participants are encouraged to formulate individual treatment goals, thereby intending to strengthen motivation. The goals are to be realistic and concrete and can be modified throughout the training. Treatment success, progress difficulties, and individual coping strategies they might face can be documented in the activity diary.

#### Behavioral activation: overcoming difficulties and pleasant activity scheduling (session 2)

The second session provides strategies to overcome difficulties concerning the active implementation of planned activities and offers input on how to reduce avoidant behaviors related to non-implementation of homework. As activities to restore congruence from session 1 might not be self-rewarding in the short-term, the second session also focuses on rather short-term mood-enhancing activities. Participants are encouraged to plan such individually chosen activities for the following week.

#### Psychoeducation (session 3)

Psychoeducational information on depression and anxiety is provided, and factors of etiology and maintenance are explained. To deepen understanding of depression and anxiety, influencing factors, risk factors, and factors of upkeep are presented. There is a short excurse on antidepressants. After a general overview, participants can choose throughout the session whether they would like to receive more detailed information on depression, anxiety, or both. Participants are encouraged to explore their previous and current symptom history, and describe the course of their symptomology.

#### Cognitive restructuring (session 4)

Participants are educated on the causal relationship between cognitions, emotions, and behavior. A thought protocol is introduced which is divided into three components: situations, thoughts, and consequences. Participants can individualize their personal thought protocols by including personal situations, cognitions, and emotions. The second phase of the thought protocol contains questioning the reality basis of negative thoughts, and intends for participants to identify positive and helpful thoughts and to generally concentrate on and practice positive thinking habits.

#### Problem solving or exposure (session 5−6)

At this stage in the training, participants can choose whether they would like to work on either problem solving or practice exposure to fear-inducing situations. In problem solving participants are educated on the distinction between solvable and unsolvable problems and they are supposed to assign their individual problems to these two categories. Following this is the concept of a 6-step problem-solving plan in which they (a) identify an individual problem, (b) define a realistic target state, (c) explore possible solutions and decide on the most promising, (d) set up a detailed solution plan, (e) execute the plan, and (f) verify the implementation process. Participants are encouraged to practice their plan the following week in real life situations. They evaluate the success or failure of the plan in the following session and can then opt to modify their plan accordingly.

Participants focusing on reducing their anxieties learn about avoidant and safety behavior, the concept of habituation through frequent confrontation of fear, and at the end of the session participants should practice what they have learned in theory, and expose themselves to fear-inducing situations. The mechanisms of exposure are explained in detail, highlighting the physical and psychological processes, with an emphasis placed upon the effectiveness of this strategy in the long run for overall well-being compared to avoidant strategies. Participants are requested to identify individual difficult or fear-inducing situations in an anxiety hierarchy. They are advised to start with their least fearful situations and to increase intensity by enhancing practice. In a challenge management protocol, participants can practice exposure in a theoretical mindset. In the next session, they evaluate the success of the implementation of their plan. They can also identify personal safety behavior and are prepared to focus on a more challenging situation which they are supposed to practice the following weeks.

#### Plan for the future (session 7)

Participants see brief content summaries in the 7th session and are asked to review the progress they have made concerning individual training goals. Participants are instructed to identify training mechanisms and strategies which helped them in reducing symptoms of depression and anxiety. They are able to write a personal letter addressed to themselves in which they assume they have completed four more weeks of practicing newly acquired strategies and can set goals for the coming 4 weeks.

#### Booster session (session 8)

In the booster session, which participants receive 4 weeks after completion of the 7th session, participants can reflect on their learning experience and individual goal attainment. They are encouraged to explore their current state of mental health and receive additional resources on support if needed. They are also able to review the letter to themselves, can reassess their goals and are instructed to make plans to implement their intentions and actions in the following months.

### Guidance and adherence monitoring

eCoaches support the participants throughout the training by providing content focused guidance and adherence reminders. Guidance can be a beneficial factor of internet interventions (51) and can have a positive effect on treatment adherence (52). eCoaches are psychologists or psychotherapists in training, licensed psychotherapists or trained masters' level psychology students. eCoaches communicate with the participants via the messaging function on the platform. The main role of the eCoach is to motivate, guide participants, and monitor adherence to the treatment as well as risk for deterioration, crisis, or other adverse effects. Within 48 h of module completion, participants receive written feedback on each completed module. Feedback is based on a standardized eCoaching manual which contains preformulated standardized text blocks prepared for each session. The feedback will be individualized for each participant and each module. As adherence is monitored, if participants fail to complete a module within 7 days (14, 21 days) they will receive individualized reminder messages by the eCoach. After 28 days reminders will be sent via text message.

### Assessments

Clinician-based and self-report assessments will take place at screening (T0), baseline (T1), post-assessment 8 weeks after randomization (T2), 6-month follow-up (T3), and 12-month follow-up in the IC group (T4). There will also be weekly mood assessments during the first seven weeks of the study phase. See Figure 1 for a detailed overview of the study flow and Table 2 for an overview of all assessments. Clinician-based diagnostic assessments will be conducted via telephone.

**Table 2:** Overview of self-report assessments.

**Construct**	**Questionnaire**	**T0**	**T1**	**T2**	**T3**
Demographics	Socio-demographic data (SozDemo)	✓	–	–	–
**PSYCHOPATHOLOGY**					
Depression	Center for Epidemiological Studies Depression Scale—Short Version (CES-D)^*^	✓	✓	✓	✓
Anxiety	General Anxiety Disorder Measurement (GAD-7)^*^	✓	✓	✓	✓
Depression	Patient Health Questionnaire (PHQ-9)	–	✓	–	–
Alcohol abuse	The Alcohol Use Disorders Identification Test (AUDIT-C)	–	✓	✓	✓
**QUALITY OF LIFE**					
	EuroQol (EQ-5D)	–	✓	✓	✓
	Assessment Quality of Life (AQoL-8D)	–	✓	✓	✓
**RESOURCE USAGE**					
	Client Service Receipt Inventory (CSRI)	–	✓	–	✓
**TRAINING**					
Expectancy	Credibility Expectancy Questionnaire (CEQ)	–	✓	–	–
Negative treatment effects	Inventory to assess negative effects of psychotherapy (INEP)	–	–	✓	–
Program evaluation	Client Satisfaction Questionnaire (CSQ-8)	–	–	✓	–
**OTHER**					
Moderators of treatment outcome	Risk Factor Questionnaire (RFQ)	–	✓	–	–
Worry	Penn State Worry Questionnaire—Ultra Brief (PSWQ-3)	–	✓	✓	✓
Sleep	One item of Pittsburgh Sleep Quality Index (PSQI)	–	✓	✓	✓
Emotion regulation	Subscales of emotion regulation scale questionnaire (SEK−15)	–	✓	–	–
Incongruence	Incongruence questionnaire—short version (INK-K)	–	✓	✓	✓
Personality	Big Five Inventory (BFI-10)	–	✓	–	–
Behavioral activation	The Behavioral Activation for Depression Scale (BADS-FS−9)	–	✓	✓	✓
Intervention drop-out reasons	Drop-Out Reasons (DG)	–	–	✓	–
Other help	Other help (INH)	–	–	✓	–

T0, screening; T1, baseline; T2, post-assessment; T3, 6-month follow-up.

* *CES-D and GAD-7 assessed weekly until post-assessment*.

Adherence to assessments completion will be monitored throughout the study. In case of noncompliance, study participants will be contacted after 7 (14, 21) days to remind them of assessment completion. After 28 days short messages will be sent via mobile phone.

#### Primary outcome

The primary outcome will be clinician-based ratings of depressive symptom severity at post-assessment (T2). Clinician-rated depressive symptom severity will be assessed by the QIDS-C (53) which covers nine criteria with 16 items: sleep, sad mood, appetite/weight, concentration/decision making, self view, thoughts of death or suicide, general interest, energy level, and restlessness/agitation. The items are each rated from 0 to 3. A meta-analysis found Cronbach's alpha to be acceptable to good (α = 0.69 − 0.89) (54). Trained psychologists' who are blind to treatment condition will conduct the diagnostic interviews. Interrater reliability will be examined by rating interviews a second time of ~10% of participants by a second rater. Several measures will be taken to ensure blinding: (1) explaining to the participant the importance of keeping the interviewers blinded to randomization status, (2) verbal reminder before each assessment, (3) documentation after each assessment whether the assessor is still blind to study condition.

#### Secondary outcomes

##### Psychopathology

Secondary outcome measures include a clinician-based change in diagnostic status (depression and anxiety), clinician-rated symptom severity of anxiety, and self-report symptom severity of anxiety and depression, the number of participants that responded and in remission, substance use, sleep quality, quality of life, and others.

Diagnostic status will be assessed at baseline, post-assessment, 6-month follow-up and 12-month follow-up (only in the IC) by sections of the MINI covering depression and anxiety (social phobia, generalized anxiety disorder, panic, agoraphobia) (48). Self-reported depression symptom severity referring to the previous week will be assessed by the Center for Epidemiological Studies Depression Scale (CES-D; 20 items, item range 0–3; reliability α = 0.95) (55, 56). The Patient Health Questionnaire (PHQ-9; 9 items; item range 0–3; reliability α = 0.89) (57– 61). The SIGH-A, a clinician-based anxiety rating, is used to assess anxiety severity (SIGH-A; 14 items; 0–4; reliability α = 0.89) (62). Anxiety is also measured in self-report by the generalized anxiety disorder measurement (GAD-7; 7 items; item range 0–3) (61).

To evaluate response and remission rates, participants will be coded as responders when they demonstrate a symptom reduction of 50% assessed by CES-D or GAD-7 respectively. Remission is defined a priori as a non-pathological score of ≤6 for the QIDS-C (53).

Other measures included alcohol consumption use (AUDIT-C; 3 item; item range 0–4 reliability α = 0**.**61) (63, 64), worrying (PSWQ-3; 3 items; item range 0–6; α = 0**.**85) (65, 66), sleep quality (PSQI; 1 item; item range 1–4; α = 0**.**74) (67, 68), behavioral activation (BADS-FS; 9 items; item range 1–6; α = 0**.**73) (69– 71), and quality of life [AQoL-8D; 35 items; item range1–5; α = [0.69–0.90] (72); EQ-5D-5L; 5 items plus 1 scale; item range 1–5; α = [0.51–0.96] (73– 75)].

##### Weekly mood assessments

Depressive and anxiety symptoms will be measured weekly with the CES-D and GAD-7 from baseline (week 1) to post-assessment (week 8). This allows a temporally more precise assessment of mood rating of the IC group compared to the WLC during the intervention phase.

##### Training acceptability and adherence

User satisfaction with the training will be assessed after the intervention phase using the Client Satisfaction Questionnaire (CSQ-8; 8 items; item range 1–4; reliability α = 0.91) (76) adapted to the online context (77). Other assessments related to the training include the credibility expectancy questionnaire (CEQ) (78, 79), and a questionnaire on negative effects of online training (adaption of Inventar zur Erfassung Negativer Effekte von Psychotherapie—INEP) (80). Adherence to session completion is tracked throughout the intervention by the training platform.

##### Resource usage

To assess the impact of the intervention on publicly-funded services and the wider society, individuals' use of services, and societal impacts such as productivity loss will be gathered by the Client Service Receipt Inventory (CSRI) adapted to the German and Swiss Health Care System (81).

#### Predictors and moderators of disorder course and treatment outcome

Predictors and moderators of treatment outcome and factors associated with the development and maintenance of MDD and anxiety will be assessed at baseline. These are based on a recent review of factors such as symptoms and other easily-assessed clinical features predicting long term treatment outcome in MDD (47), as well as a literature search [search terms in PubMed in June 2016: “risk factor” AND (“depression” OR “anxiety”)] on factors predicting onset or maintenance of MDD. Predictors and moderators of treatment outcome could help explain treatment heterogeneity (47). Predictors and moderators will be assessed in the demographic questionnaire, through the aforementioned clinical assessment instruments, and in a risk factor questionnaire (RFQ).

##### Demographic information

A demographic questionnaire will, among other sociodemographic information, assess age, gender, education, migration status, height, weight, ethnicity, current job situation, size of household, income, sexual orientation, and religiosity/spirituality.

##### Mental health

Additional mental health assessments will include 16 items from validated instruments, and cover perceived stress (82), anxiety sensitivity, (83), need for affect (84), worry (65), resilience (85), energy levels, and suicide attempts.

##### Stressful life events and trauma

Stressful childhood and lifetime events will be assessed by approximately 38 items using the Childhood Trauma Questionnaire (86), family pathology and suicidality, experiences of abuse, accident involvement, experiences of discrimination, the List of Threatening Life events (87), and job satisfaction.

##### Lifestyle

Lifestyle will be assessed by the Simple Physical Activity Scale (SIMPAQ) (88) which assesses hours of sleep, physical activity, exercise, and sedentary behavior in the previous week as well as subjective diet quality, diet satisfaction, smoking, and drug usage habits.

##### Self-image

Aspects of self-image will be covered by 17 items, including two on self-esteem (89), body satisfaction assessed by the Eating Disorder Examination (90), and perceived social and minority status.

##### Relationship satisfaction and loneliness

Relationship satisfaction will be assessed by the 10 item short version of the relationship satisfaction questionnaire (91), and loneliness will be assessed by the three item scale (92).

##### Other assessments

Other assessments will include subjective sleep quality assessed by the Pittsburgh Sleep Quality Index (PSQI) (93), chronic medical conditions, the five items of the Sense of Mastery questionnaire (94), 23 item short version of the incongruence questionnaire (95), the assessment of behavioral activation (BADS-FS) (70), the ultra-short version of the Big Five Inventory (BFI-10) (96), and various emotion regulation skill scales (subscales comprehension, acceptance, and emotional self-support by the German Emotion Regulation Scale Questionnaire, ERSQ-27, ERSQ-ES-GD) (97, 98).

### Statistical analyses

Statistical analyses will be reported in accordance with the statement by Consolidated Standards of Reporting Trials (CONSORT) (99).

#### Clinical analyses

Data analysis will be performed on an intention-to-treat (ITT) basis. Additionally, per protocol analysis including only participants who have completed the primary outcome will be analyzed. Missing data will be handled by multiple imputations (MI) with 100 iterative estimations per value, as this is currently regarded as the gold standard with regard to missing data (100). Demographic variables as well as other baseline variables will be inserted into the prediction model to estimate missing values. Differences in the primary and secondary outcomes between the two study groups will be examined with repeated measurements analysis of variance, and standardized effect sizes (Cohen's *d*) and number needed to treat (NNT) and their respective confidence intervals will be calculated for comparison. Response and remission rates will be compared across groups with contingency tables and differences tested with χ2. All unidirectional hypotheses will be tested one-sided, bidirectional hypotheses will be tested two-sided. Alpha is set to *p* < 0.05. All analyses will be conducted with SPSS.

#### Power and sample size calculation

We expect superiority of the IC compared to the WLC in the primary outcome, clinician-based depression symptom severity at post-assessment. The most recent systematic review on internet-based interventions for MDD found a standardized mean difference of *g* = −0.90, 95% CI −1.07 to −0.73 between the ICs compared to untreated controls (27). We based the sample size calculation on the lower bound of the 95% confidence interval. To demonstrate standardized effect sizes, Cohens *d*, of 0.73 with a power (1 – β) of 80% and an alpha of 0.05 in a one-sided test (calculated using G^*^Power) 48 individuals would need to be included in total (24 per group). As we aim to also explore moderators of treatment outcome, and as simulation studies show that the sample size required to detect an interaction effect of the same magnitude as in the primary analysis needs to be about three to fourfold (101), we will include *N* = 200 participants. This sample allows to detect effect sizes of *d* = 0.35 in the primary endpoint analysis.

#### Predictor and moderator analyses

Predictors and moderators of treatment outcome will be analyzed on an exploratory basis with a priori defined potential moderators: sociodemographic factors such as gender, age, income, education, ethnicity, socioeconomic status, education level, status of employment, relationship status, sexual orientation, religious, and spiritual orientation, current and previous mental health including age of MDD onset, MDD severity, low energy, number of previous episodes, duration of episodes, MDD chronicity, concurrent antidepressant use, current/past suicidality, suicide attempts, comorbid anxiety, anxiety sensitivity, substance use, perceived stress, insomnia/sleep problems, rumination, need for affect, sense of mastery, self-esteem, resilience, self-regulation, quality of life, experience with psychotherapy, physical health including diet, weight, body mass index, body satisfaction, number of medical conditions, physical functioning, sedentary behavior, smoking, drug use, quality of life, life events including childhood maltreatment/trauma, and life events, minority, and perceived social status, discrimination, experience of abuse, parental and familial psychopathology, social environment, social support/relationships, relationship satisfaction, loneliness, personality traits such as neuroticism and openness to experiences, and psychological coping skills such as emotion regulation skills. Regression analyses will be used to test interaction effects between baseline moderators and the intervention condition, using the SPSS macro PROCESS (102). Significant interactions will further be investigated applying the Johnson-Neyman technique to detect the region of significance (103).

#### Health economic evaluation

The health economic evaluation will be based on a combination of cost-effectiveness analyses (CEA) and cost-utility analyses (CUA). It will be conducted from a societal perspective including all relevant costs and a public health care perspective including only direct medical costs in a time frame of 6 months. The incremental cost-effectiveness ratio (ICER) in the CEA is expressed by the incremental costs per additional disorder free participant. The ICER in the CUA is expressed as incremental costs per quality-adjusted life years gained (QALY) based on the AQOL-8D. To test the robustness of the ICERs and to quantify uncertainty around the ICER, bootstrapping (5,000 times) will be used. The results will be plotted on a cost-effectiveness plane where the horizontal axis reflects differences in effects and the vertical axis differences in costs, and in a cost-effective acceptability curve disclosing the probability that the intervention is cost-effective for a range of willingness-to-pay ceilings. The robustness of the base-case findings will be tested by a multi-way-sensitivity analysis.

## Discussion

Even though depression and anxiety are highly comorbid, they are generally not treated by transdiagnostic treatments protocols. With the present study, we aim to provide evidence on the efficacy and cost-effectiveness of an internet- and mobile-based transdiagnostic treatment for depression and comorbid anxiety disorder and aim to explore for whom such treatments might be suitable and for whom not.

To best assess depressive symptoms at baseline and follow-ups, we will have diagnostic interviews and self-report which is highly recommended (104). To minimize potential bias in the clinical interviews we have taken precautions to ensure blinding of outcome assessors at all assessment times. As transdiagnostic treatment protocols are scarce and studies are needed to further evidence-base of the treatment potential, the study is based on a new conceptualization and integrates transdiagnostic elements targeted at depression and anxiety and combines this with individual participant tailoring offering the participants individualization options throughout the training. This method has the potential to heighten engagement, as individuals must continuously make active choices but also has the potential to heighten efficacy, as individuals choose content based on personal need.

This study also has a number of limitations. First, to not overburden the study participants we did not assess mediators. Hence, this study won't provide information about relevant mechanisms of change, which should be tested in future studies. Second, in this study we will employ an open recruitment strategy mimicking a public mental health approach, in which participants self-select to participate which can be considered a selection bias. Individuals who are familiar with the utilization of technology and the Internet will be more likely to participate (105). The results will therefore only be applicable to individuals suffering from an MDD who willingly choose to participate in internet-based treatment to improve their mood and reduce depressive symptoms. Third, although there was some involvement of individuals in the intervention development phase, the extent was limited and should be addressed more systematically and intensely in future studies (106). Fourth, although the study is powered for moderation analyses, it will not be stratified for extreme values on assessed moderator variables. Hence, it is possible that some observations on investigated moderators, e.g., very high chronicity of depression, will not sufficiently be included to detect an existing effect. And lastly, although this is one of a few studies initially powered to detect the interaction effect between a moderator and the treatment, the sample size still only allows to detect moderator effects in size range of medium to large even though smaller effects could also be of clinical interest. However, including a wide range of potential moderators determined through an extensive literature review can be a basis for future individual patient data meta-analyses, which are often at a disadvantage as primary trials commonly do not include relevant moderator variables (107, 108). Trial results will be published in a relevant peer-review journal.

Overall, to enhance evidence-based treatment options for comorbid disorders, more RCTs evaluating transdiagnostic treatments are needed. Transdiagnostic tailored treatments delivered online bear great potential to tackle the issue of comorbidities. This study will continue to build evidence for transdiagnostic treatment protocols, and might shed some light on the question for whom such treatments are suitable.

## Author contributions

DDE, KKW, and A-CZ designed the study. KKW wrote the first draft. All authors contributed feedback and approved the final manuscript.

### Conflict of interest statement

DDE and MB are stakeholder of the GET.ON Institute for Online Health Trainings (www.geton-institut.de) which aims to transfer scientific knowledge related to the present research into routine healthcare. The foundation of such an institute which disseminates research findings and products developed within research projects was the primary aim of the European Union for funding the associated research project (EU EFRE; ZW6-80119999, CCI 2007DE161PR001). The remaining authors declare that the research was conducted in the absence of any commercial or financial relationships that could be construed as a potential conflict of interest.
